# Modulation of MicroRNAs by Phytochemicals in Cancer: Underlying Mechanisms and Translational Significance

**DOI:** 10.1155/2015/848710

**Published:** 2015-03-17

**Authors:** Sanjeev K. Srivastava, Sumit Arora, Courey Averett, Seema Singh, Ajay P. Singh

**Affiliations:** ^1^Mitchell Cancer Institute, University of South Alabama, Mobile, AL 36604, USA; ^2^Department of Biochemistry and Molecular Biology, University of South Alabama, Mobile, AL 36688, USA

## Abstract

MicroRNAs (miRNAs) are small, endogenous noncoding RNAs that regulate a variety of biological processes such as differentiation, development, and survival. Recent studies suggest that miRNAs are dysregulated in cancer and play critical roles in cancer initiation, progression, and chemoresistance. Therefore, exploitation of miRNAs as targets for cancer prevention and therapy could be a promising approach. Extensive evidence suggests that many naturally occurring phytochemicals regulate the expression of numerous miRNAs involved in the pathobiology of cancer. Therefore, an understanding of the regulation of miRNAs by phytochemicals in cancer, their underlying molecular mechanisms, and functional consequences on tumor pathophysiology may be useful in formulating novel strategies to combat this devastating disease. These aspects are discussed in this review paper with an objective of highlighting the significance of these observations from the translational standpoint.

## 1. Introduction

MicroRNAs (miRNAs) are endogenous, small noncoding RNA molecules that posttranscriptionally regulate gene expression. miRNAs bind to the 3′-UTRs of target mRNA with partial or complete complementarity, thus causing translational repression or target messenger RNA (mRNA) degradation [1]. An individual miRNA can regulate the expression of multiple genes; conversely, a single mRNA can be targeted by many miRNAs. To date, about 2,469 miRNAs have been identified in humans [2], and more than one-third of all human genes are potentially regulated by miRNAs [3]. Extensive studies have shown that miRNAs not only are involved in the process of cell development and differentiation but also play a critical role in carcinogenesis [4]. Emerging data suggest that several classes of naturally occurring, plant-derived compounds (phytochemicals) could potentially regulate the expression of several miRNAs involved in cancer.

Phytochemicals are nonnutritive plant chemicals that have various applications including anti-inflammatory and anticancer. These phytochemicals are widely distributed in various fruits, vegetables, herbs, beverages, and many other dietary supplements. Numerous studies have demonstrated that the intake of fruit- and vegetable-rich foods decreases the occurrence of cancer [5–7]. So far, more than 10,000 phytochemicals have been identified [8], and a significant number of phytochemicals show anticancer potential with no or minimal toxicity to normal cells [9]. Interestingly, around 47% of FDA approved anticancer drugs are derived from plants [10, 11]. Moreover, these phytochemicals could be used as a single chemotherapeutic agent or in association with standard anticancer drugs. Phytochemicals can increase the efficacy of anticancer drugs synergistically, while reducing the toxic side effects of the standard chemotherapeutic drugs [12, 13]. These phytochemicals exert their anticancer effects through modulation of multiple molecular targets affecting various signaling pathways [8, 14, 15]. In the present review paper, we focus our attention on the regulation of miRNAs by some of the phytochemicals such as resveratrol, epigallocatechin-3-gallate (EGCG), curcumin, camptothecin (CPT), and diindolylmethane (DIM) for cancer prevention and therapy.

## 2. Biogenesis of miRNA and Mechanism of Gene Silencing

The biosynthesis of miRNAs begins with gene transcription by RNA polymerase II or RNA polymerase III into primary miRNA (pri-miRNA) transcripts inside the nucleus. These pri-miRNAs are comprised of either a cluster or single miRNAs folded into a hairpin stem-like structure [1]. This long pri-miRNA is processed by the sequential, endonucleolytic cleavage of the transcript by the microprocessor complex, containing drosha and DGCR8, into a 65–70-nucleotide precursor miRNA (pre-miRNA). This pre-miRNA is then exported from the nucleus to the cytoplasm by the nuclear export factor Exportin-5/Ran-GTP and cleaved by RNase III endonuclease, Dicer/TRBP, and argonaute 2 (Ago2) miRNA duplex [16] into an ~22-nucleotide product. This duplex miRNA then unwinds to generate a single-stranded miRNA. One of the strands enters in the RNA-induced silencing complex (RISC) along with Ago proteins and directs the complex to target mRNA. This binding causes either target mRNA degradation or inhibition of translation [1].

## 3. Biological Significance of miRNAs in Cancer: Tumor Suppressors and Oncogenes

Based on* in vitro* and* in vivo* studies, miRNAs have been characterized as tumor suppressors or oncogenes. In this section we will discuss some of the reports from a long list of tumor suppressor/oncogenic miRNAs that have been experimentally validated.

Numerous studies have shown that the expression of the tumor suppressor Let-7 is significantly downregulated or lost in various cancers [17–19]. It has been shown that restoration of Let-7 inhibited tumor growth in a K-RAS lung cancer model [20]. Furthermore, decreased expression of let-7 correlated with shorter survival in non-small-cell lung cancer [21]. Other miRNAs such as miR-15a and miR-16 are either deleted or downregulated in most cases of the chronic lymphocytic leukemia, and their overexpression induced apoptosis [22]. Moreover, it has been reported that miR-16 and miR-15 were downregulated in multidrug-resistant human gastric cancer cells, and restoration of these miRNAs sensitized the cancer cells to chemotherapeutic drugs [23]. Takeshita and coworkers demonstrated the growth inhibitory role of miR-16 in prostate cancer cells [24]. The levels of miR-34 are significantly decreased in various cancers, and its restoration was reported to inhibit angiogenesis and malignant behavior, yet at the same time it induced apoptosis and cell cycle arrest [25, 26]. Additionally, the role of miR-34 in the inhibition of tumor-initiating cells has also been suggested [27]. In mixed lineage leukemia, the overexpression of miR-495 inhibited cell viability and reduced leukemogenesis* in vivo* [28]. In one of our recent studies, we investigated the significance of miR-150 downregulation in pancreatic cancer [29]. We demonstrated that the restoration of miR-150 inhibited the MUC4 oncoprotein. Consequently, the growth and malignant potential of pancreatic cancer cells were suppressed [29, 30]. The role of miR-451 as a tumor suppressor miRNA by inhibiting cancer cell migration, invasion, and growth is well known [31–33]. Further, Kovalchuk and coworkers demonstrated that miR-451 sensitizes MCF-7/DOX-resistant cells to doxorubicin cytotoxicity [34]. Xu and coworkers demonstrated that miR-203 overexpression effectively inhibited cell proliferation and induced apoptosis and cell cycle arrest. Moreover, its overexpression also inhibited tumor growth in a mouse model [35].

To date, several miRNAs have been identified that act as oncogenes [36]. miR-17-92 is an oncogenic gene cluster [37]; restoration of this cluster in MYC-driven B-cell lymphomas suppressed apoptosis and enhanced tumorigenicity [37]. Ma et al., 2010, demonstrated that miR-27a, which is overexpressed in pancreatic cancer, plays an oncogenic role by targeting tumor suppressor Spry2 [38]. Inhibition of miR-27a decreased growth, clonogenicity, and migration of pancreatic cancer cells. miR-21 is a widely studied oncogenic miRNA which is frequently overexpressed in various malignancies [39, 40]. Inactivation of miR-21 results in apoptosis induction, inhibition of growth, and malignant progression [39–41]. In a transgenic mice model, miR-155 induced polyclonal pre-B-cell proliferation resulting in B-cell leukemia [42]. miR-373 and miR-520c are known to promote migration, invasion, and metastasis of breast cancer cells by targeting CD44 expression [43]. Moreover, the roles of miR-373 and miR-520c in promoting migration and invasion of prostate cancer cells have also been reported [44]. In pancreatic cancer, miR-424-5p is overexpressed, and its high expression has been reported to be associated with enhanced proliferation and apoptosis resistance through downregulating SOCS6 [45]. miR-10b is overexpressed in various malignancies and promotes cell migration, invasion, and metastasis [46–48]. Moreover, a high expression of miR-10b correlated with disease progression [47, 49]. miR-221 and miR-222 are known to facilitate tumor cell growth, malignant potential, and EMT in multiple malignancies such as prostate, breast, and thyroid cancer [50–53].

Moreover, overexpression of miR-221 and miR-222 was shown to impart tamoxifen-resistance in breast cancer cells [54]. Altogether, these findings indicate that miRNAs play important roles as oncogenes in cancer cells.

## 4. Modulation of miRNAs by Phytochemicals

miRNAs are being considered as attractive targets for cancer prevention and therapy due to their oncogenic or tumor suppressor activities. Various studies have suggested that the modulation of miRNAs serves as one of the key mechanisms in the anticancer activities of a variety of phytochemicals (Table 1). Below, we describe some of the phytochemicals which are known to regulate miRNA expression in cancer.

### 4.1. Resveratrol

Resveratrol is a stilbenoid that has been shown to have anticancer activities against various cancers including breast cancer, lung cancer, glioma, prostate cancer, colon cancer, and neuroblastoma [55, 56]. Resveratrol reduced the expression of numerous oncogenic miRNAs, namely, miR-17, miR-21, miR-25, miR-92a-2, miR-103-1, and miR-103-2, in human colon cancer cells [57]. Moreover, in the same study, tumor suppressor miR-663 levels were shown to be restored in human colon cancer cells after the treatment of resveratrol. In another study, Hagiwara et al. reported that resveratrol treatment upregulated miR-141 and resulted in a significant reduction of invasiveness, whereas resveratrol-induced miR-200c expression caused reversal of EMT through downregulation of Zeb1 and upregulation of E-cadherin [58]. It has been demonstrated that the anticancer effect of resveratrol on pancreatic cancer cells was due to inhibition of oncogenic miR-21 [59]. Moreover, the synergistic antitumor activity of resveratrol and miR-200c has been demonstrated in human lung cancer cells [60]. In colon cancer cells, resveratrol inhibited the cell growth and induced apoptosis through upregulating miR-34a expression [61].

### 4.2. Epigallocatechin-3-gallate (EGCG)

Epigallocatechin-3-gallate is a polyphenol flavonoid that possesses significant antioxidant and anticancer properties. It has been shown that EGCG induces apoptosis in hepatocellular carcinoma through enhanced expression of miR-16 [62]. Increased expression of miR-16 resulted in inhibition of its target antiapoptotic Bcl-2, followed by mitochondrial dysfunction, cytochrome c release, and subsequent apoptosis. EGCG also inhibited the expression of miR-21 followed by repression of androgen receptor (AR) signaling and, consequently, a reduction of prostate cancer cell growth [63]. In lung cancer, EGCG upregulated the expression of miR-210, which led to the inhibition of proliferation and anchorage-independent growth [64]. EGCG enhanced the efficacy of cisplatin through downregulation of miR-98-5p in A549 non-small lung cancer cells [65]. A combination of N-(4-hydroxyphenyl) retinamide and EGCG decreased the expression of oncogenic miRs (miR-92, miR-93, and miR-106b) and enhanced the expression of tumor suppressor miRs (miR-7-1, miR-34a, and miR-99a) which resulted in growth inhibitory effects in human malignant neuroblastoma cells [66].

### 4.3. Genistein

Genistein is another important polyphenol that showed significant anticancer effects through the regulation of miRNAs. Genistein treatment was shown to enhance apoptosis synergistically with miR-16 in human chronic lymphocytic leukemia cells [67]. In a study on prostate cancer, genistein both downregulated miR-221 and miR-222 and restored tumor suppressor gene aplasia Ras homolog member I (ARHI) expression, which ultimately resulted in anticancer effects [68]. In another study on prostate cancer, genistein inhibited the migration and invasion of PC3 and DU145 cells through downregulating oncogenic miR-151 [69]. Xu et al. have shown that treatment of ovarian cancer cells with genistein caused an inhibition of cell growth and migration through suppression of miR-27a [70]. Further, genistein has been shown to upregulate the tumor suppressor miR-574-3p in prostate cancer cells [71]. Moreover, it has been observed that genistein exerted its antitumor effect in prostate cancer via downregulation of miR-1260b [72]. Genistein treatment downregulated oncogenic miR-1260b and resulted in inhibition of Wnt-signalling in renal cancer cells [72]. miR-223 expression was found to be downregulated in pancreatic cancer cells after genistein treatment that correlated with cell growth inhibition and induction of apoptosis [73]. Genistein also plays a tumor suppressor role through inhibition of miR-27a in pancreatic cancer cells [74].

### 4.4. Curcumin

Curcumin is a constituent of turmeric (*Curcuma longa*) and has been used as an important component of spice in Indian food and as a traditional medicine in Asian countries for many decades [75]. It possesses chemopreventive and chemotherapeutic activities against many tumors [75–78]. Curcumin exerts its therapeutic effects by regulating miRNAs known to play an important role in cancer [79]. Yang et al. have shown that curcumin upregulated miR-15a and miR-16 in MCF-7 breast cancer cells which caused an induction of apoptosis [80]. In another study, curcumin treatment resulted in the upregulation of tumor suppressor miR-203 in bladder cancer that led to apoptosis induction and diminished proliferation, migration, and invasion [81]. Curcumin has also been shown to induce tumor suppressor miR-186∗ expression to promote apoptosis in lung cancer [82]. Moreover, curcumin inhibited the transcriptional regulation of oncogenic miR-21 in colon cancer, causing inhibition of growth, invasion, and metastasis [83]. Zhao et al. provided evidence that curcumin exerts its cytotoxic effects against SKOV3 ovarian cancer cells largely through upregulation of miR-9 [84]. Another tumor suppressor, miR181b, has been demonstrated to be induced by curcumin, and it inhibited breast cancer metastasis via downregulation of the inflammatory cytokines CXCL1 and CXCL2 [85]. High levels of miR-221 expression have been correlated with shorter survival in pancreatic cancer patients, suggesting that miR-221 could be an oncogenic miRNA [86]. In the same study, the synthetic curcumin analogue (CDF) has been found to suppress the expression of miR-221 and upregulate the expression of PTEN, p27 (kip1), p57 (kip2), and PUMA, followed by inhibition of cell proliferation and migration of pancreatic cancer cells. Thus, altogether, these studies provide evidence that curcumin modulates the expression of miRNA signatures in cancer cells to confer its anticancer activity.

### 4.5. Quercetin

Intake of a quercetin-rich diet has been demonstrated to modulate the expression of 48 unique miRNAs. These miRNAs have been reported to decrease tumor metastasis and invasion (miR-146a/b, 503, and 194), inhibit cell proliferation (miR-125a, 155, let-7 family, 302c, 195, 26a, 503, and 215), induce apoptosis (miR-125a, 605, 26b, let-7g, 34a, 491, and 16), and upregulate tumor suppressor miRNAs (let-7 family, miR-125a, 183, 146a, 98, 19b, 106a, and 381) [87]. Del Follo-Martinez et al. reported that quercetin treatment induced apoptosis in colorectal cancer cells when used along with resveratrol. The underlying mechanism of apoptosis induction is the downregulation of oncogenic miR-27a [88]. In another study, quercetin, when used with catechins, was shown to enhance the expression of let-7 in pancreatic cancer cells followed by K-ras inhibition and reduction of the advancement of pancreatic cancer [89].

### 4.6. Camptothecin (CPT)

Camptothecin, an alkaloid isolated from bark of* Camptotheca acuminata*, is a potent chemotherapeutic agent against a variety of tumors [90–92]. CPT was demonstrated to reduce the expression of miR-125b significantly, which led to the upregulation of Bak1 and p53 and resulted in apoptosis of human cervical cancer and myelogenous leukemia cells [92]. In a recent study, camptothecin was shown to inhibit HIF-1*α* by enhancing the levels of miR-155, miR-17-5p, and miR-18a in HeLa cells [93].

### 4.7. Diindolylmethane (DIM)

Diindolylmethane is an active compound that is generated in the stomach through the metabolic conversion of indole-3-carbinol (I3C), present in cruciferous vegetables [94]. DIM regulates the expression of numerous miRNAs involved in cancer development and progression. DIM has been demonstrated to induce the expression of certain miRNAs, such as miR-200 and let-7 families, that led to the reversal of EMT and enhanced chemosensitivity in gemcitabine-resistant pancreatic cancer cells [95]. DIM also induced the expression of miR-146a, which resulted in reduced pancreatic cancer cell invasion via inhibition of metastasis-associated protein 2 (MTA-2), interleukin-1 receptor-associated kinase 1 (IRAK-1), and NF*κ*B [96]. Moreover, Jin observed that DIM inhibited breast cancer cell growth by enhancing the expression of miR-21 which led to the degradation of its target Cdc25A [97]. Formulated 3,3′-diindolylmethane (BR-DIM) has been shown to be capable of downregulating miR-221, resulting in growth inhibition of pancreatic cancer cells [86].

## 5. Mechanism of miRNA Regulation by Phytochemicals

**Figure 1:**
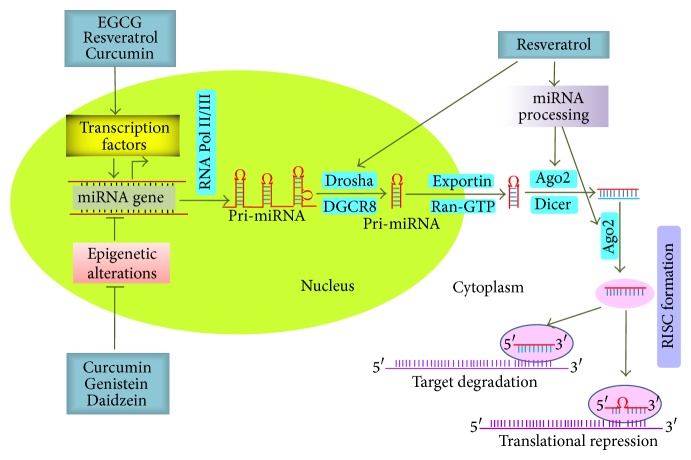
Mechanistic overview of miRNA regulation by phytochemicals. Phytochemicals regulate the expression of miRNAs through modulation of transcription factors and inducing epigenetic modifications and by interfering with processes associated with miRNA maturation.

There have been several aberrantly expressed miRNAs identified in various cancer types. Unfortunately, the precise mechanisms that regulate the normal expression of miRNAs or their deregulation in cancer remain unclear. Growing evidence suggests that aberrant transcriptional regulation, epigenetic changes, alterations in miRNA biosynthesis machinery, mutations, or DNA copy number could contribute to miRNA dysregulation in human cancer [98]. Experimental studies on phytochemicals revealed that the expression of various miRNAs can be regulated by phytochemicals. In the following section, we provide an overview for the regulation of miRNAs by phytochemicals through various mechanisms such as epigenetic, transcriptional, and miRNA processing (Figure 1).

### 5.1. Transcriptional Regulation of miRNAs

Various studies indicate the involvement of certain transcription factors in the regulation of miRNA expression and the subsequent modulation of pathological conditions in cancer. Thus, transcription factor-mediated miRNA regulation is one critical aspect of study. Several groups have observed that phytochemicals modulate miRNA expression through transcriptional regulation (Figure 1). Wang and coworkers demonstrated that treatment with EGCG results in the transcriptional activation of miR-210 in lung cancer by promoting the binding of hypoxia inducible factor-1*α* (HIF-1*α*) to the hypoxia response element present in the promoter region of miR-210 [64]. Transcription factor activator protein (AP-1), an important regulator of genes involved in cell proliferation and extracellular matrix production [99], is an upstream regulator of miR-21 [100]. Mudduluru and coworkers demonstrated that curcumin inhibits the transcriptional regulation of miR-21 by downregulating AP-1 to suppress tumor growth, invasion, and metastasis of colorectal cancer [83]. Another transcription factor, CCAAT/enhancer binding protein beta (C/EBP-*β*), negatively regulates tumor suppressor miR-145 by directly interacting through the putative C/EBP-*β* binding site present in the miR-145 promoter. It was also shown that resveratrol treatment resulted in the decreased activation of C/EBP-*β* which subsequently induced the expression of miR-145 in breast cancer cells [101]. Androgen receptor (AR) directly regulates miR-21 by binding to the miR-21 promoter in prostate cancer [102]; Siddiqui and coworkers (2011) demonstrated that EGCG inhibited prostate cancer cell growth by decreasing the level of AR and miR-21 [63]. A study conducted by Hagiwara and coworkers has shown that resveratrol transcriptionally upregulated the expression of several tumor suppressor miRNAs such as miR-141, miR-26a, miR-195, miR-126, miR-340, miR-34a, miR-193b, miR-335, miR-200c, miR-497, miR-196a, and miR-125a-3p in MDA-MB-231 breast cancer cells [58]. It is known that p53 is a tumor suppressor gene and a transcription factor which functions by either causing growth arrest or inducing apoptosis [103]. Emerging data suggest that p53 transcriptionally regulates several miRNAs [104]. A number of studies have shown that curcumin regulates the expression of several miRNAs that are transcriptional targets of p53 such as miR-22, miR-15/16a, miR-34, and miR-21 [80, 83, 105–108]. Considering all these observations, it is speculated that curcumin regulates the expression of p53, which in turn modulates the expression of several miRNAs.

### 5.2. Epigenetic Regulation of miRNAs

Epigenetic regulation is defined as modifications of the genome without any change to the nucleotide sequence. Supporting evidence suggests that epigenetic modification, like aberrant CpG methylation or histone modifications, contributes to the dysregulation of gene expression in tumor cells. Pharmacologic inhibition of DNA methyltransferase (DNMT) causes DNA demethylation and upregulates the expression of miRNAs. Saini and coworkers provided evidence that curcumin treatment resulted in the hypomethylation of miR-203 promoter and subsequent upregulation of miR-203, which is epigenetically silenced in various malignancies [81]. Treatment with a curcumin analogue, difluorinated curcumin, restored the expression of miR-34a and miR-34c by working as a demethylating agent in colon cancer cells [109]. Rabiau and coworkers performed miRNA expression profiling following the treatment with the flavonoids genistein and daidzein in prostate cancer cells [110]. Their investigation revealed a significant upregulation of miR-548b-5p in PC3 and miR-15a in LNCaP cells by genistein and daidzein through regulation at the epigenetic level [110]. Zaman et al. demonstrated that miR-145 is inactivated as a result of its promoter methylation in prostate cancer; treatment with genistein demethylated the promoter of miR-145 which resulted in an increased level of miR-145 [111]. In a separate study on prostate cancer, isoflavone efficiently demethylated the promoter region of miR-29a and miR-1256 and subsequently upregulated their expression [112]. All these observations suggest the significant role of phytochemicals in the regulation of miRNAs at the epigenetic level (Figure 1).

### 5.3. Regulation of miRNA Processing by Phytochemicals

miRNA processing can be regulated at various steps, and any alteration in processing either increases or decreases the level of miRNAs [113]. The role of phytochemical-modulated expression of miRNAs through the regulation of proteins involved in miRNA processing has been recently investigated, as shown in Figure 1. Hagiwara and coworkers investigated and reported that resveratrol treatment significantly increased the expression of Ago2 and results in enhanced levels of tumor suppressor miRNAs such as miR-16, miR-141, miR-143, and miR-200c in MDA-MB-231 cells [58]. Moreover, resveratrol treatment resulted in the enhanced level of miR-663 and pre-miR-663 by interfering with the drosha mediated processing of pri-miR-663 that resulted in the inhibition of miR-155, which is overexpressed in many cancers [114]. These studies provide evidence that phytochemicals regulate the expression of miRNAs epigenetically and transcriptionally and by controlling miRNA processing.

## 6. Conclusion and Future Perspective

miRNAs are a novel class of gene regulators. Thus far, we have only been able to see just a small part of the complexity of cellular regulation by miRNAs with regard to all of the different functions that miRNAs can perform. miRNAs are aberrantly expressed in most cancers and this has been correlated throughout cancer initiation and progression; hence, miRNAs represent very attractive and novel targets for cancer therapy. Phytochemicals display an inimitable ability to alter the level of miRNAs involved in regulation of cancer pathobiology by modulating the expression of miRNAs through a variety of mechanisms, namely, epigenetic, transcriptional, and miRNA processing. In addition, phytochemicals increase the chemosensitivity of conventional therapeutic drugs through modulating miRNAs. As a result, phytochemicals can be exploited for designing therapeutic approaches in combination with conventional therapies to improve cancer treatment and prevention strategies. Despite the potent anticancer activities of phytochemicals, there are some concerns such as specific targeting and bioavailability. To overcome these hurdles in the use of phytochemicals, different approaches are being explored, namely, chemical modification, synthetic formulation, delivery by nanoparticles, and so forth. Hence, there is a wide scope for the development of phytochemicals into commercial drugs to efficaciously prevent and treat cancer.

## Figures and Tables

**Table 1:** Phytochemical-mediated regulation of miRNAs in cancer. Modulation of miRNA expression by certain phytochemical agents and effect on cancer pathobiology.

Phytochemical	miRNA	Function	Reference
Resveratrol	↑ miR-141, miR-663, and miR-200c	Invasiveness, EMT, and metastasis	[57–61, 114]
	↓ miR-17, miR-21, miR-25, miR-92a-2, miR-103-1, and miR-103-2		

EGCG	↑ miR-16, miR-210, miR-7-1, miR-34a, and miR-99a	Apoptosis	[62–66]
	↓ miR-21, miR-98-5p, miR-92, miR-93, and miR-106b	Proliferation, anchorage-independent growth, and drug resistance	

Genistein	↑ miR-16	Apoptosis	[67, 68]
	↓ miR-221 and miR-222	Growth	

Curcumin	↑ miR-15a, miR-16, and miR-186^*^	Apoptosis	[80, 82, 83]
	↓ miR-21	Metastasis	

Quercetin	↑ let-7	Growth Apoptosis	[88, 89]
	↓ miR-27a		

Camptothecin	↓ miR-125b	Apoptosis	[92]

DIM	↑ miR-21, miR-200b, miR-200c, let-7b, let-7c, let-7d, let-7e, and miR-146a	Growth, EMT, drug resistance, invasion, and metastasis	[95–97]
